# The role of ALOX15 in inflammation-related diseases

**DOI:** 10.3389/fimmu.2026.1790402

**Published:** 2026-04-02

**Authors:** Rong Tang, Chao-yang Gong, Yang Liu, Hai-lian Zhong, Ying-bin Wang, Hai-yu Zhou

**Affiliations:** 1 Gansu Province Key Laboratory of Bone and Joint Diseases, Lanzhou, China; 2 The Second Hospital of Lanzhou University, Department of Anesthesiology, Lanzhou, China; 3 The Second Hospital of Lanzhou University, Department of Orthopedics, Lanzhou, China

**Keywords:** ALOX15, inflammation resolution, inflammatory diseases, inflammatory response, lipid mediators

## Abstract

Arachidonate 15-lipoxygenase (ALOX15) is a key member of the lipoxygenase family, catalyzing the oxidation of polyunsaturated fatty acids (PUFAs) to produce numerous biologically active lipid mediators. Recent studies have revealed that ALOX15 and its metabolites play a complex regulatory role in inflammatory responses. They are not only involved in inflammation resolution through the production of pro-resolving mediators but can also generate pro-inflammatory lipid signals that exacerbate inflammatory damage. Research utilizing gene knockout and transgenic animal models further indicates that ALOX15 contributes to the pathogenesis of various inflammation-related diseases, including neuroinflammation, atherosclerosis, asthma, rheumatoid arthritis, and metabolic inflammatory diseases. This article systematically reviews the current understanding of the role of ALOX15 in inflammation-associated diseases.

## 1 Introduction

Chronic inflammation-related diseases, such as atherosclerosis, asthma, inflammatory bowel disease (IBD), and rheumatoid arthritis (RA), constitute a major global health burden. Their continuously rising incidence and disability rates place a significant strain on healthcare systems (1). The common core of these diseases lies in the failure of the body’s inherent inflammatory response to be terminated in a timely and effective manner, thereby transforming from a protective process into sustained tissue damage (2). In this complex pathological process, lipid mediators derived from polyunsaturated fatty acids (PUFAs) play a crucial role. Acting as precise chemical messengers, they are not only responsible for the initiation and amplification of inflammation, more importantly, govern the active resolution of inflammation and the initiation of tissue repair. Their metabolic balance directly determines the progression and outcome of the inflammatory response (3–5).

Arachidonic acid (AA) is an essential PUFAs and serves as the primary precursor for eicosanoids, which in addition to their roles in various physiological functions, are implicated in numerous diseases, including atherosclerosis, diabetes, and neurological disorders. AA is metabolized through three major enzymatic pathways: cyclooxygenase (COX), lipoxygenase (LOX), and cytochrome P450 monooxygenase (cytochrome P450) (6, 7). Consequently, a wide range of molecules targeting the bio-oxidation process of AA have been developed into clinical therapeutics. Among them, the COX pathway, targeted by classical nonsteroidal anti-inflammatory drugs (NSAIDs) and primarily responsible for generating mediators such as prostaglandins and thromboxanes, has long been a focal point in anti-inflammatory treatment (8). In parallel, the LOX pathway, predominantly governed by the lipoxygenase family, has gained increasing recognition for its biological significance.

Lipoxygenases (LOXs) are non-heme iron-containing dioxygenases that catalyze the stereospecific peroxidation of PUFAs, generating a series of bioactive lipid mediators. Typical products include various hydroperoxy PUFAs and leukotrienes (LTs). These lipid mediators primarily regulate the responsiveness, proliferation, and differentiation capacities of various cell types, playing significant roles in anti-infection, oncology, and inflammation, among other processes (9). In humans, there are six known functional LOX genes (ALOX5, ALOX12, ALOX12B, ALOX15, ALOX15B, ALOXE3), as shown in **Table 1**. They are conventionally designated “ALOX” for arachidonate lipoxygenases. All ALOX genes are sequentially arranged within a gene cluster without overlapping each other (10).

**Table 1:** Human LOX isozymes, their substartes, products and tissue distribution.

Human LOXs	Main substrates	Main products	Tissue distribution	References
AL0X5	Arachidonic acid	5(S)-HpETE	Granulocytes, monocytes/macrophages, mast cells, dendritic cells and B lymphocytes	(11)
AL0X12	Arachidonic acid	12(S)-HpETE	Platelets and their precursors, megakaryocytes, keratinocytes and tumor cells	(12)
AL0X12B	Arachidonic acid	12(R)-HpETE	Skin and hair follicles	(13)
	Linoleic acid	9(R)-HpODE		(14)
AL0X15	Arachidonic acid	15(S)-HpETE 12(S)-HpETE	Immune cells and epithelial cells	(15)
	Linoleic acid	13(S)-HpODE		(16)
	Docosahexaenoic acid	17(S)-HpDHA	Corneal epithelium and brain tissue	(17)
AL0X15B	Arachidonic acid	15(S)-HpETE	Hair roots, prostate, lungs, skin and corneas	(18)
	Linoleic acid	13(S)-HpODE		(19)
AL0XE3	Arachidonic acid	12(R)-HpETE	Epithelial cells of the skin, tongue and stomach	(3)
	Linoleic acid	9(R)-HpODE		(20)

HpETE, Hydroperoxyeicosatetraenoic acid; HpODE, Hydroxydocosahexaenoic acid; HpDHA, Hydroperoxydocosahexaenoic Acid.

Among these, ALOX15 has garnered particular attention due to its unique substrate preference and product diversity. It mediates the dehydrogenation and subsequent oxygenation of AA to produce both 15-hydroperoxyeicosatetraenoic acid (15-HpETE) and 12-HpETE in a ratio of approximately 9:1 (21), which can be further metabolized into lipoxins (LX), hepoxilins (HX) and eoxins (EX) with diverse biological effects (22, 23). However, the functional properties of ALOX15 exhibit marked species-specific differences that must be considered when interpreting preclinical data. While human ALOX15 predominantly oxygenates AA to 15-HpETE, the murine ortholog (often termed leukocyte-type 12-LOX) primarily generates 12-HpETE (24, 25). This divergence in catalytic specificity, driven by targeted enzyme evolution during primate development (10), has profound implications for the translational relevance of findings obtained from conventional rodent models and necessitates careful consideration when extrapolating animal data to human pathophysiology.

Importantly, in-depth research has revealed a thought-provoking central paradox: ALOX15 can exhibit both pro-inflammatory and anti-inflammatory effects in inflammatory diseases. On one hand, it has been reported to exacerbate the progression of diseases such as atherosclerosis and asthma through mechanisms like oxidizing low-density lipoprotein (LDL) and generating pro-inflammatory lipids (15, 26);On the other hand, due to its capacity to synthesize pro-resolving mediators, it demonstrates clear protective roles in conditions like ischemic brain (27, 28).In summary, the relationship between ALOX15 and inflammation-related diseases is highly complex. This seemingly “contradictory” dual role precisely reflects the context-dependent functionality of ALOX15, making it a key challenge and hotspot for understanding precise inflammation regulation and developing novel therapeutic strategies.

Therefore, this review aims to systematically delineate the spectrum of roles played by ALOX15 across various inflammation-related diseases, delve into its functions and microenvironmental determinants, as well as comprehensively evaluate the potential and challenges of targeting ALOX15 and its downstream pathways as a therapeutic strategy, thereby providing a theoretical foundation for the development of novel treatment paradigms.

## 2 Biological characteristics and inflammatory regulatory functions of ALOX15

### 2.1 Enzymatic basis and key metabolites of ALOX15

**Figure 1:**
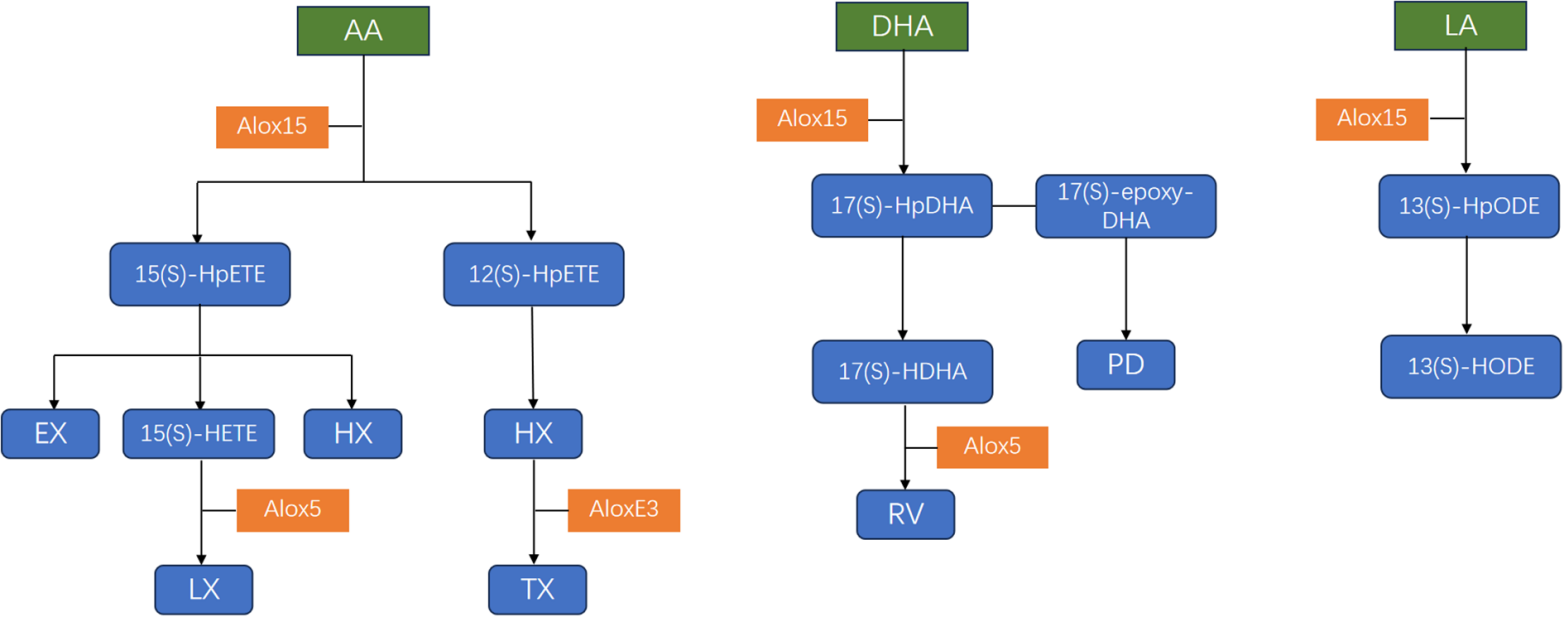
Fatty acid substrates and metabolites of human ALOX15. ALOX15 metabolizes arachidonic acid (AA) to generate 12(S)-HpETE and 15(S)-HpETE, which are further converted to their respective hydroxides, 12(S)-HETE and 15(S)-HETE, respectively. 12(S)-HpETE, 15(S)-HpETE and 15(S)-HETE can also be converted to other lipid mediators such as lipoxins (LX), hepoxillins (HX), trioxillins (TX), eoxins (EX). Docosahexanoic acid (DHA) is metabolized by ALOX15 into 17(S)-hydroperoxydocosahexanoic acid (HpDHA), which is further metabolized to form resolvins (RV) and protectin Ds (PD). ALOX15 metabolizes linoleic acid (LA) to generate 13(S)-hydroperoxyoctadecaenoic acid (13(S)-HpODE), which is subsequently converted to 13(S)-hydroxyoctadecaenoic acid (13(S)-HODE).

ALOX15 is constitutively expressed in reticulocytes, eosinophils, dendritic cells, macrophages, and epithelial cells (29, 30). Its expression is regulated by multiple factors including cytokines (IL-4, IL-5, and IL-13), hypoxia, oxidative stress, and epigenetic modifications (31–33). ALOX15 possesses a C-terminal domain essential for its catalytic activity and an N-terminal β-barrel domain. It is generally localized in the cytoplasm, but can undergo membrane translocation in the presence of factors such as Ca^2+^, significantly enhancing its apparent enzymatic activity (34). The primary physiological substrates of this enzyme are AA, linoleic acid (LA) and docosahexaenoic acid (DHA) (35). Details are shown in **Figure 1**.

It primarily catalyzes the formation of 15(S)-HpETE and 12(S)-HpETE from AA. These products are subsequently reduced by cellular glutathione peroxidases to their corresponding hydroxyl analogues, namely 15(S)-HETE and 12(S)-HETE (22). 15(S)-HpETE and/or 15(S)-HETE can be further metabolized into various bioactive products, such as lipoxins (LXs), hepoxillins (HXs), and eoxins (EXs). LXA4, LXB4, aspirin-triggered (AT)-LXA4, and AT-LXB4 are a class of anti-inflammatory mediators that contribute to resolving inflammatory responses and inflammatory diseases in animal models (35). HX isomers, such as HXA3 and HXB3, are involved in regulating inflammatory responses and insulin secretion (36). EXs (EXC4, EXD4, and EXE4) exhibit pro-inflammatory effects and have been implicated in severe asthma and allergic reactions (37). 12(S)-HpETE and/or 12(S)-HETE bind to and activate G protein-coupled receptor 31 (GPR31) and the BLT2 (38).They can also be metabolized to HXA3 and HXB3, which are subsequently converted into their respective trihydroxy metabolites, such as trioxilin A3 (TXA3) and TXB3. These metabolites induce vasodilation, promote pain perception, reverse oxidative stress, and stimulate insulin secretion in various animal model systems (39).

ALOX15 can also efficiently catalyzes DHA, generating various lipid mediators with important biological functions. Initially, ALOX15 oxidizes DHA to 17S-hydroperoxy-DHA (17S-HpDHA), with a catalytic efficiency comparable to that for AA, confirming DHA as an effective physiological substrate of this enzyme (17). Subsequently, 17S-HpDHA can further serve as a substrate for ALOX15, undergoing a second oxidation reaction to form the key intermediates 17S-epoxy-DHA and 17(S)-hydroxy-DHA (17S-HDHA). Among these, 17S-epoxy-DHA has been confirmed as a direct precursor in the biosynthesis of protectin D (PD), while 17-hydroxy-DHA is considered a critical intermediate in the resolvins (RV) synthesis pathway (40). These products play key roles in inflammation resolution, immune regulation, inhibition of platelet aggregation, and modulation of synaptic plasticity (17).

In addition, ALOX15 can also catalyze LA to produce 13(S)-hydroperoxyoctadecadienoic acid (13-HpODE) and 13-HpETE (41). Studies have shown that 13(S)-HpODE binds to PPAR-delta, reduces its activation and expression, thereby promoting apoptosis in colorectal cancer cells (16). In conclusion, the relationship between ALOX15 and inflammation-related diseases is complex and context-dependent. Positioned at a critical node in PUFA metabolism, ALOX15 generates downstream products with both pro-inflammatory and anti-inflammatory activities, which underpins its dual role in pathology.

### 2.2 Pro-inflammatory effects of ALOX15

A growing body of research has confirmed the pro-inflammatory effects of ALOX15 and its metabolites (42, 43). Firstly, they have the capacity to directly recruit and activate inflammatory cells. Both 12-HETE and 15-HETE act as potent chemokines that directly attract and activate neutrophils, eosinophils, dendritic cells, macrophages, and mast cells. These compounds not only facilitate the recruitment and infiltration of these cells into sites of inflammation but also enhance the adhesive properties and degranulation processes of inflammatory cells, thereby significantly amplifying the inflammatory response (4, 44). For instance, studies have shown that ALOX15 promotes the migration of eosinophils and neutrophils to sites of inflammation, which is a key feature in the progression of chronic rhinosinusitis with nasal polyps (45, 46). Overexpression of ALOX15 in airway epithelial cells can induce the release of chemokines such as macrophage inflammatory protein-1α/β (MIP-1α/β), regulated on activation, normal T cell expressed and secreted (RANTES), and interferon gamma-induced protein 10 (IP-10), thereby significantly promoting the chemotaxis of immature dendritic cells (30). This recruitment of antigen-presenting cells is crucial, as it bridges the innate and adaptive immune responses. The resulting influx of dendritic cells, together with the direct recruitment of granulocytes, creates a microenvironment that favors the development of Th2 responses. This amplifies the production of IL-4, and form a positive feedback loop that locks the tissue into a state of persistent type 2 inflammation (47).

Additionally, 12-HETE and 15-HETE can activate the mitogen-activated protein kinase (MAPK)/extracellular signal-regulated kinase (ERK) signaling axis, triggering the phosphorylation and activation of a series of downstream transcription factors. This ultimately drives the upregulation of gene expression for key pro-inflammatory cytokines such as tumor necrosis factor-α (TNF-α), interleukin-1β (IL-1β), and interleukin-6 (IL-6), as well as chemokines like monocyte chemoattractant protein-1 (MCP-1) (48). On the other hand, these lipid mediators can also target the nuclear factor kappa B (NF-κB) pathway by promoting the phosphorylation and degradation of its inhibitory protein (IκBα) or activating the upstream IκB kinase (IKK) complex. This leads to the dissociation and nuclear translocation of NF-κB transcription factor family members, thereby broadly inducing the expression of numerous genes involved in inflammation, cell survival, and proliferation (43, 49). This synergistic activation of central signaling pathways enables ALOX15-derived products to efficiently integrate and amplify inflammatory signals from various intracellular and extracellular stimuli, forming a powerful positive feedback loop. This mechanism plays a crucial role in the initiation and persistence of chronic inflammatory diseases.

**Figure 2:**
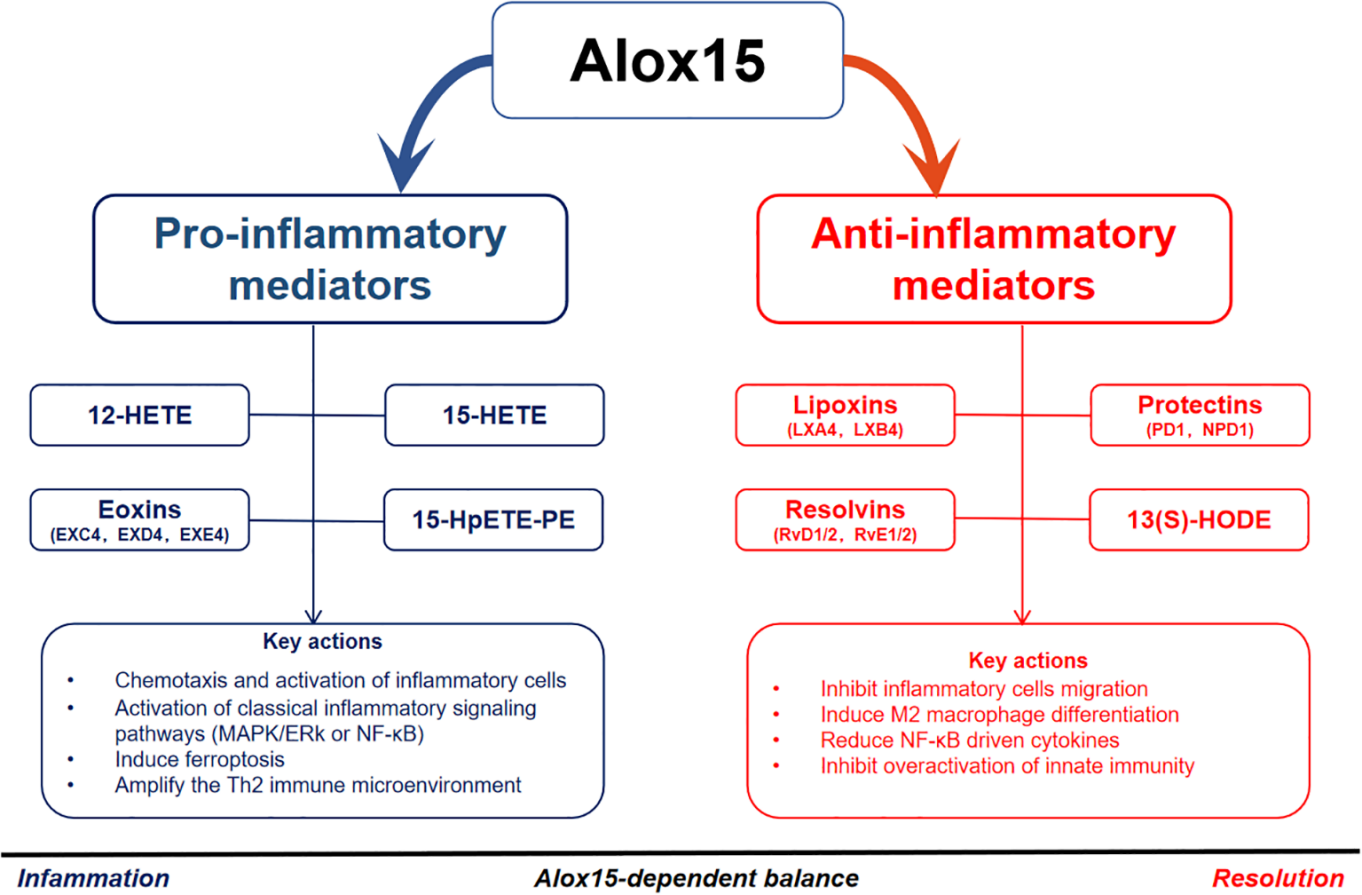
ALOX15 and its key metabolites in inflammation versus resolution. Pro-inflammatory mediators produced by Alox15 include 12-HETE, 15-HETE, 15-HpETE-PE, and eoxins (EXC4, EXD4, EXE4), which promote chemotaxis, activation of inflammatory cells, MAPK/ERK or NF-κB signaling, ferroptosis, and Th2 immune amplification. Anti-inflammatory mediators such as lipoxins (LXA4, LXB4), resolvins (RvD1, RvD2, RvE1, RvE2), protectins (PD1, NPD1), and 13(S)-HODE inhibit inflammatory cell migration, induce M2 macrophage differentiation, reduce NF-κB-driven cytokines, and suppress innate immunity overactivation. The balance between these opposing mediators determines the outcome of inflammation (progression vs. resolution). 12-HETE, 12-hydroxyeicosatetraenoic acid; 15-HETE, 15-hydroxyeicosatetraenoic acid; 15-HpETE-PE, 15-hydroperoxyeicosatetraenoic acid-phosphatidylethanolamine; Alox15, arachidonate 15-lipoxygenase; EXC4, eoxin C4; EXD4, eoxin D4; EXE4, eoxin E4; LXA4, lipoxin A4; LXB4, lipoxin B4; RvD1, resolvin D1; RvD2, resolvin D2; RvE1, resolvin E1; RvE2, resolvin E2; PD1, protectin D1; NPD1, neuroprotectin D1; 13(S)-HODE, 13(S)-hydroxyoctadecadienoic acid; MAPK, mitogen-activated protein kinase; ERK, extracellular signal-regulated kinase; NF-κB, nuclear factor kappa B; Th2, T helper type 2; M2, alternatively activated macrophages.

More importantly, recent studies emphasize that ferroptosis is also recognized as a significant mechanism in inflammation (50). The expression of ALOX15 and related enzymes (such as PEBP1) promotes lipid peroxidation and the production of 15-hydroperoxyeicosatetraenoic acid-phosphatidylethanolamine (15-HpETE-PE), a key ferroptosis marker, which enhances inflammation by amplifying the activation of inflammatory pathways (51). The study by Yang et al. (52) demonstrated that the Th2-type cytokine IL-13 induces ferroptosis in bronchial epithelial cells by inhibiting the expression of glutathione peroxidase 4 (GPX4) and SLC7A11, thereby reducing glutathione (GSH) levels. This process not only leads to the accumulation of lipid peroxides but also triggers the release of inflammatory cytokines (IL-1β, IL-6, and TNF-α) and reactive oxygen species (ROS) from epithelial cells, directly driving airway inflammation. Building on this, Kagan’s team revealed that ALOX15-mediated lipid peroxidation is closely associated with acute asthma exacerbations, leading to the hypothesis that ALOX15-regulated ferroptosis in bronchial epithelial cells may underlie this phenomenon (53). This concept establishes a mechanistic link between ALOX15 enzymatic activity and IL-13-triggered ferroptosis. Shah et al. (54) demonstrated that ferroptosis cells potently activate macrophages, prompting the release of substantial pro-inflammatory mediators. Concurrently, the lipid peroxides and arachidonic acid-derived inflammatory mediators generated during ferroptosis exacerbate the inflammatory cascade. Synthesizing these findings, a coherent mechanistic axis emerges: IL-13 activates ALOX15 and inhibits GPX4, leading to ferroptosis in bronchial epithelial cells, which in turn activates macrophages and releases mediators, thereby perpetuating airway inflammation. The key metabolites and mechanisms involved in pro-inflammatory responses are summarized in **Figure 2** and **Table 2**.

**Table 2:** Key ALOX15-derived lipid mediators, their chemical structures, receptors, and biological functions.

Metabolite	Chemical Structure	Substrate	Primary Receptor(s)	Biological Function	References
Pro-inflammatory mediators					
15-HETE	C20H32O3 (Structure: 20:4, 15-OH)	AA	PPAR-γ (partial agonist)	Activates inflammatory signaling; Chemotaxis of immune cells.	(7)
12-HETE	C20H32O3 (Structure: 20:4, 12-OH)	AA	GPR31	Chemotaxis of immune cells; Activation of MAPK/ERK pathway; Platelet aggregation.	(38)
EXC4	C30H47N3O9S (Containing glutathione group)	AA	CysLT1 receptor	Smooth muscle contraction; Vascular permeability; Pro-inflammatory cytokine release.	(37)
EXD4	C25H40N2O6S	AA	CysLT1 receptor	Involved in Th2-type inflammation.	(37)
EXE4	C22H36O6S	AA	CysLT1 receptor	Pro-inflammatory effects in eosinophilic disorders.	(37)
15-HpETE-PE	Phosphatidylethanolamine esterified 15-HpETE	AA-PE	membrane disruption	Executes ferroptosis by disrupting membrane integrity.	(53)
Anti-inflammatory mediators					
LXA4	C20H32O5 (Structure: 5S,6R,15S-triHETE)	AA	FPR2/ALX	Inhibits neutrophil chemotaxis; Promotes macrophage efferocytosis; Reduces NF-κB activation.	(82)
LXB4	C20H32O5 (Structure: 5S,14R,15S-triHETE)	AA	BLTs	Inhibits immune cell migration; Anti-inflammatory effects.	(83)
RvD1	C22H32O5 (Structure: 7S,8R,17S-triHDoHE)	DHA	GPR32(human), FPR2/ALX (murine)	Limits neutrophil infiltration; Enhances macrophage phagocytosis; Promotes tissue regeneration.	(84)
RvD2	C22H32O5	DHA	GPR18	Reduces excessive neutrophil recruitment; Protects from sepsis.	(84)
RvE1	C20H30O5 (Structure: 5S,12R,18R-triHETE)	EPA	ChemR23, BLT1 (antagonist)	Reduces inflammation; Promotes resolution; Organ protection.	(64)
PD1/NPD1	C22H34O4 (Structure: 10R,17S-diHDoHE)	DHA	GPR37	Neuroprotection; anti-apoptotic; Promotes tissue regeneration; Reduces oxidative stress.	(85)
13(S)-HODE	C18H32O3 (Structure: 18:2, 13-OH)	LA	PPAR-γ	Activates anti-inflammatory gene expression; Promotes M2 macrophage polarization.	(86)

HETE, Hydroxyeicosatetraenoic acid; HpETE, Hydroperoxyeicosatetraenoic acid; HODE, Hydroxyoctadecadienoic acid; PE, Phosphatidylethanolamine; AA, Arachidonic acid; DHA, Docosahexaenoic acid; LA, Linoleic acid; EPA, Eicosapentaenoic Acid; PPAR-γ, Peroxisome proliferator-activated receptor gamma; GPR31, G protein-coupled receptor 31; CysLT1, Cysteinyl leukotriene receptor 1; FPR2/ALX, Formyl peptide receptor 2/Lipoxin A4 receptor; GPR32, G protein-coupled receptor 32; GPR18, G protein-coupled receptor 18; ChemR23, Chemerin receptor 23; BLT1, Leukotriene B4 receptor 1; GPR37, G protein-coupled receptor 37.

### 2.3 Anti-inflammatory effects of ALOX15

Inflammation resolution is a precisely regulated process aimed at actively restoring tissue homeostasis (55). Its core lies in the fundamental transformation of cellular components and inflammatory mediator patterns within the inflammatory tissue (22). ALOX15 plays an indispensable role in this process, and its anti-inflammatory properties are realized through multiple synergistic mechanisms.

Firstly, studies have shown that ALOX15 is one of the key enzymes in the synthesis of specialized pro-resolving mediators (SPMs), including resolvins (RVs) and protectins D (PDs) (56–59). These mediators act as “stop signals” for the inflammatory response, which can reduce leukocyte migration (60), promote the apoptosis of pro-inflammatory neutrophils (61), induce the differentiation of highly efficient M2 macrophages (62), and inhibit T-cell migration and activation, thereby actively terminating inflammation and initiating tissue repair (63). For instance, in murine models of acute inflammation, the administration of RVs, particularly RvE1, significantly reduces the infiltration of neutrophils (64). In a mouse model of postoperative ileus, ALOX15 deficiency leads to decreased synthesis of PDs, resulting in increased neutrophil influx (65). And PD1 can block T-cell migration, reduce TNFα and interferon-γ secretion, and promote T-cell apoptosis (66). However, a balanced interpretation of these pro-resolving mechanisms requires caution and several lines of evidence challenge the notion that these mediators are key endogenous molecules for inflammation resolution. From a biosynthetic perspective, the classic SPM cascade has been questioned by *in vitro* enzymology studies, which indicate that many LXs and RVs are not efficiently produced from their precursors by human lipoxygenases, suggesting their *in vivo* generation may be very limited (67, 68). Furthermore, the identity and signaling of proposed G-protein-coupled SPM receptors have not been consistently validated in knockout mouse studies, and in humans, SPM levels have not been robustly linked to dietary precursor supplementation or consistently observed during the natural resolution phase of inflammation (69–71). More fundamentally, the accurate detection and quantification of SPMs in complex biological matrices presents a formidable analytical challenge. A hallmark of SPMs is that their reported concentrations are far lower than those of pro-inflammatory mediators, and reliable quantification is hampered by their instability and the limitations of current liquid chromatography-tandem mass spectrometry (LC-MS/MS) methodologies (71). A recent critical evaluation argued that commonly used analytical approaches often fail to apply standard limit-of-detection (LOD) and limit-of-quantitation (LOQ) criteria, leading to the potential misidentification of background noise as genuine SPM signals and casting doubt on the very occurrence of many of these lipids in biological samples (72). These questions regarding the formation, signaling, and reliable quantification of SPMs collectively challenge their definitive role as primary endogenous mediators of resolution. Therefore, although exogenous SPMs or the modulation of ALOX15 activity show promising therapeutic potential, future research must urgently address the aforementioned controversies. In particular, efforts should focus on validating the endogenous functions of SPMs at physiologically relevant concentrations and delineating the specific contributions of SPM-dependent versus SPM-independent pathways in the pro-resolving actions of ALOX15.

Secondly, 13S-H(p)ODE, a major product of linoleic acid catalyzed by ALOX15, also exhibits anti-inflammatory activity (73), and can suppress inflammatory gene expression by activating peroxisome proliferator-activated receptor gamma (PPARγ) signaling pathway (74). Studies have shown that stimulating linoleic acid metabolism can induce the production of 13(S)-HODE in colonic epithelial cells. It can be subsequently converted by 13(S)-HODE dehydrogenase to 13-Oxo-ODE, which binds to PPARγ and reduces IL-8 secretion, thereby exerting an anti-inflammatory effect (75). In a cerebral ischemia model, 13(S)-HODE exerts neuroprotective effects by upregulating PPARγ protein levels, promoting its nuclear translocation and DNA binding to activate this pathway, consequently inhibiting the production of key pro-inflammatory mediators such as NF-κB, iNOS, and COX-2 (76). Beyond individual metabolites, ALOX15 generates a broader array of lipid mediators that act in concert. In a murine skin wound model, injury-induced ALOX15 expression in macrophages and stem cells produces monohydroxy oxylipins that collectively activate PPARγ. Alox15 deletion caused excessive collagen deposition, persistent pro-inflammatory gene expression (IL-6, IL-1β, Cxcl2, miR-21), and impaired PPARγ/adiponectin signaling, indicating loss of PPARγ’s anti-inflammatory brake on NLRP3 and TGF-β pathways. Reconstitution with a physiological oxylipin mixture restored normal healing and PPARγ activation, demonstrating that ALOX15-generated oxylipins synergistically suppress inflammation via PPARγ (77). Therefore, The ALOX15-PPARγ axis thus represents a critical node in inflammation resolution. Multiple ALOX15-derived oxylipins—including 13(S)-HODE and wound-induced monohydroxy lipids—serve as endogenous PPARγ ligands that cooperatively suppress NF-κB, promote alternative macrophage activation, and enhance tissue repair (74, 76, 77). This positions ALOX15 as a metabolic gatekeeper converting pro-inflammatory fatty acids into anti-inflammatory signals. Therapeutically, enhancing these endogenous ligands—rather than targeting ALOX15 directly—may offer a nuanced approach to resolving inflammation while preserving homeostatic functions.

Thirdly, ALOX15 itself serves as a canonical marker of alternative (M2) macrophage polarization in humans. In response to Th2 cytokines IL-4 or IL-13, human macrophages upregulate ALOX15 expression as part of a broader transcriptional program that promotes tissue repair and inflammation resolution (29, 78). This induction is mediated by STAT6 and can be further potentiated by efferocytosis through liver X receptor (LXR) activation, which integrates sterol metabolism with anti-inflammatory gene expression (78). Functionally, ALOX15 in M2 macrophages contributes to the biosynthesis of SPMs precursors such as 15-HETE and 17-HDHA, linking macrophage phenotype to the production of pro-resolving lipid signals. ALOX15 activity also influences cholesterol homeostasis and membrane remodeling, processes critical for effective efferocytosis and tissue repair (44). In the tumor microenvironment, ALOX15 signaling has been implicated in the polarization of tumor-associated macrophages (TAMs) toward an immunosuppressive M2-like phenotype, contributing to lymphoma progression (79). Conversely, in high-altitude hypoxic lung injury, downregulation of ALOX15 promotes M2 polarization and alleviates ferroptosis and inflammation (80). These findings underscore that the functional outcome of ALOX15 expression in macrophages is highly context-dependent. Understanding the regulatory networks that control ALOX15 expression and activity in specific disease settings will be essential for harnessing its therapeutic potential.

Furthermore, specific oxidized phospholipids generated via ALOX15 catalysis can inhibit the overactivation of innate immunity by blocking Toll-like receptor binding, thereby participating in the inflammation resolution program (81). Studies have found that when the concentration of oxidized phospholipids is 10-fold lower than that required to induce a pro-inflammatory response, they can potently suppress the upregulation of lipopolysaccharide-induced inflammatory cytokines (81). This demonstrates that some distinct effects of the same metabolites may be attributed to their varying concentrations.

Therefore, although ALOX15 exhibits pro-inflammatory activity during the early phase of inflammation, it plays a crucial anti-inflammatory and tissue-protective role during the resolution phase through multiple pathways, including the generation of pro-resolving mediators, activation of anti-inflammatory signaling pathways, and production of regulatory oxidized lipids. This underscores its complex yet pivotal dual role in the dynamic equilibrium of inflammation. The key metabolites and mechanisms involved in anti-inflammatory responses are summarized in **Figure 2** and **Table 2**.

## 3 The pathophysiological role of ALOX15 in inflammation-related diseases

### 3.1 Neuroinflammatory diseases

#### 3.1.1 Ischemic cerebrovascular disease

Numerous studies have demonstrated the role of ALOX15 in ischemic cerebrovascular disease (87, 88). Its involvement spans the entire disease process and exhibits an evolving “double-edged sword” characteristic over time. In the early phase of ischemia, increased release of AA and elevated intracellular calcium in the brain facilitate membrane binding and activation of ALOX15 (89). In a mouse model of transient middle cerebral artery occlusion, van Leyen et al. demonstrated that ALOX15 was significantly upregulated in the penumbra surrounding the core infarct, a brain region susceptible to ischemia-induced delayed cell death (87). At this stage, the mechanisms involve both direct neuronal injury and vascular disruption. As shown in the study by Zhang et al. (90), peroxynitrite triggers intracellular zinc release, which activates ALOX15, leading to ROS accumulation, p38 MAPK and caspase-3 activation, and ultimately causing mitochondrial dysfunction and neuronal death. In parallel, Jin et al. demonstrated that ALOX15 upregulation in endothelial cells results in degradation of the tight junction protein claudin-5, increased IgG extravasation, and exacerbate edema by compromising blood–brain barrier integrity (91). They are even associated with increased levels of the pro-apoptotic factor AIF, directly participating in cell death pathways within the ischemic area (92, 93).Conversely, functional inactivation of the ALOX15 gene protects mice from stroke (94), reduces blood-brain barrier leakage and edema formation (91), and also mitigates post-stroke behavioral deficits (95). Pretreatment of animals with ALOX15 inhibitors can replicate these protective effects. For instance, in a mouse model of transient middle cerebral artery occlusion, intraperitoneal administration of baicalein protects against ischemia-reperfusion injury by inhibiting ALOX15 pathway-mediated neuronal cell death (87). Which further confirms the early pro-injury role of ALOX15.

However, as the disease progresses into the subacute phase, a significant functional transformation occurs in ALOX15, with its derived mediators (including LXs and PDs) assume a pivotal role in neuroprotection and the promotion of inflammation resolution (22). For example, in rats with permanent middle cerebral artery occlusion, rosiglitazone not only induces the expression of LOX in the brain but also increases LXA4 levels while inhibiting the production of LTB4, thereby exerting neuroprotective effects (96). Additionally, in a rat model of ischemic stroke, activation of the LXA4 receptor effectively limits cortical inflammatory damage by inhibiting microglial activation and reducing neutrophil infiltration and recruitment, subsequently reducing infarct volume, alleviating vasogenic edema, and preserving blood-brain barrier integrity (97). Furthermore, neuroprotectin D1 (NPD1) reduces tissue damage in animal stroke models by downregulating ischemia-reperfusion injury-induced leukocyte infiltration, pro-inflammatory signaling and infarct size (98). Since ALOX15 participates in the synthesis of NPD1 from docosahexaenoic acid (DHA), this enzyme is likely involved in this protective effect as well. Interestingly, administration of DHA after focal cerebral ischemia in rats promotes neurobehavioral recovery, reduces cerebral infarction and edema, and activates the synthesis of NPD1 in the penumbra (99). In summary, ALOX15 plays a dynamic and central regulatory role in cerebral ischemia. This dualistic and temporal nature makes it a highly promising, yet requiring precise intervention, therapeutic target for the treatment of cerebral ischemia.

#### 3.1.2 Alzheimer’s disease

Alzheimer’s disease (AD) is a chronic neurodegenerative disorder characterized by progressive memory loss. Oxidative stress and neuroinflammation play significant roles in its pathogenesis (100). Multiple clinical observations have demonstrated the involvement of ALOX15 in the onset and progression of AD. For instance, a study by Pratico et al. reported elevated levels of ALOX15 and its metabolites 12(S)-HETE and 15(S)-HETE in the frontal and temporal brain regions of AD patients (101); Yao et al. also found increased levels of 12- and 15-HETE in the cerebrospinal fluid of AD patients (102). This observation was consistent with the activation of ALOX15 during amyloid-beta (Aβ) peptide-induced primary neuronal cell death *in vitro*. Inhibition of ALOX15 activity using specific inhibitors or antisense oligonucleotides effectively blocked the cytotoxicity, thereby implying a pro-injury role for ALOX15 in this process (103, 104). Furthermore, a series of studies have revealed that ALOX15 not only regulates Aβ plaque production and tau phosphorylation but also modulates AD-associated synaptic pathology and behavioral deficits (105, 106). This perspective was further supported by evidence from transgenic AD mouse models, where genetic deletion or functional inhibition of ALOX15 significantly reduced Aβ plaque generation and deposition, ameliorated tau pathology, and alleviated memory dysfunction (107, 108). However, it was noteworthy that some reports indicated that the decrease of ALOX15 expression in the hippocampus of AD patients was correlated with a reduction in its protective metabolite NPD1 (109). This discrepancy may reflect influences from different disease stages, brain region specificity, or the complexity of AD pathology.

#### 3.1.3 Parkinson’s disease

Parkinson’s disease (PD) is a neurodegenerative disorder characterized by the degeneration and loss of midbrain dopaminergic neurons and a decrease in striatal dopamine, leading to clinical features such as bradykinesia, resting tremor, and cogwheel rigidity. ALOX15 also plays a significant role in PD (110). An early and highly specific decline in substantia nigra glutathione (GSH) is associated with PD, with low GSH levels causing degeneration in cultured dopaminergic neurons. Li et al., using immature cortical neurons and cloned neuronal cell lines, demonstrated that GSH depletion triggered the activation of neuronal ALOX15. This lead to peroxide production, Ca^2+^ influx, and ultimately cell death. Importantly, inhibition of AA metabolism or treatment with ALOX15 inhibitors could block GSH depletion-induced cell death (111). It has also been proposed that nitric oxide (NO) exerts both neurotrophic and neurotoxic effects on dopamine (DA) function in primary midbrain cultures. Under conditions of GSH depletion, the neurotrophic role of NO shifts to neurotoxicity, triggering programmed cell death with features of both apoptosis and necrosis (112). Subsequently, the same group demonstrated that the metabolism of AA via the ALOX15 pathway was central to the GSH-NO interaction. The metabolic product 12-HpETE could induce cell death, and its neuronal toxicity was greatly potentiated by GSH depletion. ALOX15 inhibitors (nordihydroguaiaretic acid and baicalein) were shown to prevent NO-induced neurotoxicity (113).

Furthermore, ferroptosis has garnered recent attention in PD research, which is primarily characterized by iron metabolism-mediated lipid peroxidation and GSH depletion, often accompanied by inflammatory responses (114). While initial evidence demonstrated that butylphthalide alleviates motor deficits in PD rat models by inhibiting oxidative stress and ferroptosis in the midbrain substantia nigra (115), subsequent mechanistic studies have pinpointed lipoxygenases as key drivers of this process. Kagan VE et al. suggested that ALOX15 was involved in activating PUFAs and membrane phospholipid peroxidation, and inhibiting ALOX15 expression could reduce excessive lipid accumulation in cells, thereby mitigating ferroptosis (53). Extending this concept to related family members, Li et al. demonstrated that targeting ALOX5 prevented ferroptosis in dopaminergic neurons and significantly improved behavioral deficits in PD mouse model (53, 116). This finding suggests that other lipoxygenase family members may also contribute to ferroptosis in the context of PD. While ALOX15 is thought to directly initiate phospholipid peroxidation on membranes, ALOX5 may exert its pro-ferroptotic effects through the generation of specific lipid mediators or via distinct cellular localization (24). The convergence of both ALOX15 and ALOX5 pathways on ferroptosis highlights the complexity of lipid metabolism in neuronal cell death and underscores the need to investigate the specific roles of each isoform in different cellular contexts or disease stages. Collectively, these data suggested that investigating the axis of lipid metabolism activation, which lead to lipid peroxidation and ferroptosis, might offer new therapeutic prospects for PD.

#### 3.1.4 Neuropathic pain

Neuropathic pain (NP), a chronic pain condition, results from damage or disease affecting the nervous system. The pathogenesis of NP involves multiple aspects, including neuroinflammation, activation of glial cells, dysfunction of ion channels, and involvement of autoimmune disorders (117). Recent studies have found that ferroptosis participates in the progression of NP induced by chronic constriction injury (CCI), and inhibiting ferroptosis can alleviate CCI-induced pain hypersensitivity (118). Subsequent mechanistic investigations have pinpointed ALOX15 as a central mediator linking ferroptosis to neuroinflammation in NP. Lei et al. (119) discovered that silencing the Alox15 gene could upregulate GPX4 expression, thereby reducing ferroptosis and inflammatory responses. This positions ALOX15 as a critical node integrating oxidative stress and neuroinflammation in pain pathways. Upstream regulators of ALOX15 have recently been identified, revealing multiple signaling axes that converge on this lipoxygenase. Yang et al. (120) first confirmed that modulating the SAT1/Alox15 signaling axis could decrease lipid peroxidation, inhibit ferroptosis, and alleviate skeletal muscle contusion. Based on this foundation, Wan et al. (121) further observed dysregulation of SAT1 and Alox15 gene markers in the CCI-induced NP model, providing a direct link between this axis and neuropathic pain. Moreover, electroacupuncture stimulation effectively alleviated CCI-induced pain as well as ferroptosis-related symptoms in the dorsal root ganglia, including lipid peroxidation and iron overload. Knocking down SAT1 also reduced mechanical and thermal pain hypersensitivity and reversed ferroptosis-related damage. These findings indicate that electroacupuncture stimulation treats NP by inhibiting ferroptosis through the regulation of the SAT1/ALOX15 pathway. Parallel work has identified an additional upstream pathway involving microglial ALOX15 activation. A study has shown that intrathecal administration of the lipopolysaccharide active ingredient KDO2-lid A can directly activates Toll-like receptor 4 (TLR4) in the spinal cord, thereby causing significant tactile abnormal pain and simultaneously increasing ALOX15-mediated HXs production in spinal microglia. While these effects could be completely prevented by pre-treatment with an Alox15 inhibitor or a selective antibody targeting rat ALOX15. Similarly, inhibition of spinal ALOX15 with nordihydroguaiaretic acid prevented the second-phase paw withdrawal response in the formalin test and reversed formalin-induced persistent tactile allodynia. These results indicate that the heightened pain state mediated by spinal TLR4 is at least partially achieved through the activation of microglial ALOX15 (98).

Collectively, these studies have focused on a core pathogenic mechanism: upstream signals (SAT1, TLR4) activate ALOX15, leading to lipid peroxidation and ferroptosis, which in turn trigger neuroinflammation and hyperalgesia. Therapeutic interventions targeting this mechanism, including electroacupuncture stimulation, drug inhibition of ALOX15 or regulation of upstream regulatory factors, are expected to be used in the treatment of NP.

### 3.2 Cardiovascular inflammatory diseases

#### 3.2.1 Atherosclerosis

ALOX15 participates in the initiation and progression of atherosclerosis through multiple pathways. With regard to endothelial dysfunction, its metabolite 15(S)-HETE can disrupt endothelial barrier function by activating signaling pathways such as Src, Pyk2, and PKCϵ, leading to phosphorylation of tight junction proteins (e.g., ZO-1, ZO-2) and their dissociation from occludin. Which increases vascular permeability and promotes monocyte/macrophage adhesion and migration (122–124). This process can be alleviated by ALOX15 knockout or xanthine oxidase inhibitors in high-fat diet-induced models (125).In terms of lipoprotein oxidative modification, ALOX15 can directly oxidize low-density lipoprotein (LDL), facilitating its uptake by macrophage scavenger receptors (e.g., SR-A, CD36) and thereby promoting foam cell formation (44, 126). The enzyme also oxidizes high-density lipoprotein (HDL), impairing its cholesterol reverse transport capacity and weakening its anti-inflammatory functions (127–129). The oxidation of HDL further leads to the accumulation of unesterified cholesterol within macrophages, combined with reduced cholesterol efflux, significantly contributes to the formation of foam cells and the development of atherosclerosis (130).Furthermore, ALOX15 and its metabolites (15(S)-HETE, 13(S)-HODE) upregulate the expression of the scavenger receptor CD36 in macrophages via pathways such as PPARγ and ROS/Syk/Pyk2/STAT1, thereby promoting foam cell formation (131, 132).Simultaneously, they degrade the cholesterol transporter ABCG1 through the p38 MAPK/JNK2 pathway, collectively contributing to intracellular lipid accumulation (133).In contrast to these findings, a small number of studies have reported that 13(S)-HODE activates PPARα, leading to increased ABCA1 expression and promoting macrophage cholesterol efflux, suggesting that ALOX15 may also exert anti-atherosclerotic effects (134, 135). Regarding the recruitment of inflammatory cells, ALOX15 and its metabolites enhance the expression of adhesion molecules such as MCP-1, ICAM-1, and VCAM-1 via activation of protein kinase Cα and the NF-κB pathway, while also promoting IL-17A production. These mechanisms accelerate the recruitment and adhesion of monocytes to the intima, thereby exacerbating atherosclerosis (136–139).

However, findings from animal studies revealed conflicting roles of ALOX15. In ApoE−/− or LDL receptor-deficient mice, genetic ablation of Alox15 consistently attenuates high-fat diet-induced atherosclerotic lesions, supporting a pro-atherogenic function (136, 140). In contrast, studies in transgenic rabbit models or Alox15-overexpressing mice have suggested an opposing, anti-atherogenic role, attributed to the generation of 13(S)-HODE or pro-resolving lipid mediators such as LXA4, RvD1, and PD1 (141, 142). These discrepancies likely stem from fundamental differences in lipoprotein metabolism between species, the stage of lesion development examined, and the cellular context of ALOX15 expression—factors that critically influence the balance between pro-inflammatory and pro-resolving metabolite profiles.

This apparent contradiction may reflect the dual nature of ALOX15 activity: while its products can promote oxidative damage and inflammation in established plaques, constitutive expression in healthy vasculature might contribute to homeostatic functions such as resolution of low-grade inflammation. Alternatively, the cellular source of ALOX15 (e.g., infiltrating macrophages vs. resident vascular cells) and the local lipid substrate availability could determine its net effect on plaque progression.

Human studies also showed inconsistencies. Although elevated ALOX15 expression and its characteristic metabolite 13(S)-HODE were often detected within plaques (108), certain genetic studies indicated that higher ALOX15 expression might be associated with a lower risk of disease (143). This apparent contradiction may reflect the dual nature of ALOX15 activity: while its products can promote oxidative damage and inflammation in established plaques, constitutive expression in healthy vasculature might contribute to homeostatic functions such as resolution of low-grade inflammation. Alternatively, the cellular source of ALOX15 (e.g., infiltrating macrophages vs. resident vascular cells) and the local lipid substrate availability could determine its net effect on plaque progression.

In summary, these findings underscore that ALOX15 exerts context-dependent, dual effects in atherosclerosis. Its ultimate impact is shaped by a complex interplay of cell type, disease stage, metabolic microenvironment, and the balance of its enzymatic products. Future studies employing cell-type-specific knockout models, longitudinal analyses across disease stages, and comprehensive lipidomic profiling are needed to dissect these multifaceted roles and to determine whether therapeutic targeting of ALOX15 should aim for inhibition, activation, or pathway-specific modulation.

#### 3.2.2 Myocardial ischemia-reperfusion injury

ALOX15 primarily exerts a detrimental role in myocardial ischemia-reperfusion injury (MIRI), and its pathological mechanisms involve multiple aspects. First, this enzyme catalyzes the production of hydroxy fatty acid metabolites, such as 12-HETE, 15-HETE, and 13-HODE, accompanied by substantial generation of ROS. Which aggravates oxidative stress and lipid peroxidation in cardiomyocytes, leading to damage in cell membranes and mitochondrial function (144, 145). Meanwhile, ALOX15 metabolites can upregulate the expression of adhesion molecules and chemokines, promote the infiltration of monocytes/macrophages, and amplify the local inflammatory response through pathways such as activation of the NLRP3 inflammasome, thereby exacerbating cardiac injury (146). Recent evidence indicated that apoptosis and necrosis occur in the early phase of MIRI, whereas ferroptosis becomes the predominant form of cell death during prolonged reperfusion. Researchers have demonstrated that ALOX15 expression was specifically increased in the injured area and colocalized with cardiomyocytes. Specific knockout of Alox15 alleviated ischemia-reperfusion injury in mice and restored cardiac function. 15-HpETE was recognized as a trigger for ferroptosis in cardiomyocytes, which promoted the binding of Pgc1α to the ubiquitin ligase ring finger protein 34, leading to its ubiquitin-dependent degradation. The specific Alox15 inhibitor ML351 could elevate Pgc1α protein levels, inhibit cardiomyocyte apoptosis, protect the injured myocardium, and promote the recovery of cardiac function (147). Therefore, targeting and inhibiting ALOX15 has emerged as a potential therapeutic strategy. However, its cell-specific effects, temporal window of action and interactions with other lipoxygenases still require further investigation.

### 3.3 Respiratory inflammatory diseases

#### 3.3.1 Asthma

Chronic airway inflammation is the fundamental characteristic of asthma. Analyzing molecular pathways related to airway inflammatory components and their association with asthma severity will provide crucial and transformative insights into the clinical features of the disease and potential treatment responses (148). Among these networks, bioactive lipid mediators have emerged as central regulators of the inflammatory continuum—capable of both propagating and resolving airway inflammation (149). The pro-inflammatory arm of lipid metabolism in asthma is well-established. Cysteinyl leukotrienes are significantly elevated in the bronchoalveolar lavage fluid of asthma patients, stimulate bronchial smooth muscle contraction, and act as potent chemoattractants for eosinophils and basophils (150). The clinical relevance of this pathway is underscored by the widespread use of montelukast, a cysteinyl leukotriene receptor antagonist, as adjunctive therapy for asthmatic airway inflammation (151). More recently, ferroptosis has been implicated in asthma pathogenesis. The Kagan team’s long-standing investigation into lipid peroxidation revealed that ALOX15-mediated lipid peroxidation is associated with acute asthma exacerbations, potentially through the induction of ferroptosis in bronchial epithelial cells (51, 152). These findings collectively position lipid peroxidation and its enzymatic drivers as key amplifiers of airway inflammation.

However, a more complex picture has emerged from recent studies revealing that ALOX15 and its lipid mediators also execute critical protective functions. Surprisingly, ALOX15-derived metabolites—particularly SPMs such asMaR1 and RvD1—potently inhibit eosinophilic airway inflammation at nanomolar concentrations, with eosinophils and pleural macrophages serving as primary cellular effectors of this protective activity (153, 154). This duality positions ALOX15 at the crux of inflammatory fate decisions: its enzymatic output can either fuel (via pro-inflammatory HETEs and ferroptosis) or suppress (via SPMs) airway inflammation depending on cellular context, substrate availability, and disease stage.

The recognition of this dual role is driving a fundamental shift in asthma therapeutics—from a purely “anti-inflammatory” paradigm toward a “pro-resolving” strategy. Rather than broadly suppressing lipid metabolism, future interventions may seek to selectively channel ALOX15 activity toward the production of pro-resolving mediators or to combine conventional anti-inflammatories with SPM analogs to actively promote resolution. Thus, a deeper mechanistic understanding of how the ALOX15 pathway is skewed toward either pro-inflammatory or pro-resolving outputs holds the key to developing next-generation therapies that not only halt inflammation but actively restore airway homeostasis.

#### 3.3.2 Chronic obstructive pulmonary disease

ALOX15 also plays a crucial role in the pathological progression of chronic obstructive pulmonary disease (COPD). Under stimulation by key COPD inducers such as cigarette smoke, bacterial lipopolysaccharide, and inflammatory cytokines, the expression of ALOX15 is significantly upregulated. Its metabolites directly attack and disrupt the integrity of cell membranes and mitochondrial membranes, thereby irreversibly driving cells toward ferroptosis (155). The massive death of bronchial and alveolar epithelial cells resulting from ferroptosis directly weakens the physical barrier function and repair capacity of the airways (156). Furthermore, lipid peroxides and their subsequent metabolites generated via ALOX15 catalysis are themselves potent pro-inflammatory mediators and chemoattractants, capable of persistently amplifying inflammatory circuits. This vicious cycle between ALOX15-driven ferroptosis and chronic inflammation collectively contributes to the hallmark pathological features of COPD, including damage ciliary epithelium of the airway, hyperplasia of goblet cells and excessive mucus secretion, destruct the alveolar walls (emphysema formation), and irreversible small airway fibrosis (15).

Recent studies have revealed the anti-ferroptotic effects of two natural products and their mechanisms in alleviating COPD. Firstly, sea buckthorn extract could inhibit lipid peroxidation production and GSH depletion, mitigate cellular ferroptosis and neutrophil chemotactic migration, improve the pathological phenotype of COPD induced by LPS/porcine pancreatic elastase in mice, and suppress inflammatory cytokine production. Mechanistically, it worked by directly scavenging ROS and targeting the inhibition of NADPH oxidase 4, thereby suppressing the overactivation of the p53 and MAPK signaling pathways, ultimately regulating the expression of key ferroptosis-related molecules such as SAT1, ALOX15, GPX4, and SLC7A11 (157). Secondly, scutellarein is a naturally occurring flavonoid with established antioxidant and anti-inflammatory properties. Which also significantly alleviated Ras-selective lethal small molecule 3 (RSL3)-induced ferroptosis and mitochondrial damage in bronchial epithelial cells, and improved COPD pathological changes in mice induced by LPS/cigarette smoke. Its mechanism of action is reflected in two aspects: Firstly, it reduces the level of free iron within cells by directly chelating Fe^2+^; Secondly, scutellarein exerts its effects by binding to ALOX15 and inhibiting its enzymatic activity, thereby reducing lipid peroxidation reactions, maintaining the expression of GPX4, and suppressing the excessive activation of the Nrf2/HO-1 and JNK/p38 signaling pathways (155).

These findings not only deepen the understanding of ferroptosis in COPD but also indicate that ALOX15, as a pivotal molecular nexus linking oxidative stress, lipid metabolism dysregulation, ferroptosis, and chronic inflammation, represents a highly promising therapeutic intervention target in the pathogenesis and progression of COPD.

### 3.4 Autoimmune diseases

#### 3.4.1 Rheumatoid arthritis

In the complex pathophysiology of rheumatoid arthritis (RA), ALOX15 emerges as a central pathogenic node that integrates inflammatory signaling with metabolic dysregulation. Expressed predominantly in synovial macrophages, fibroblasts, and subsynovial endothelial cells within the RA synovium (158). ALOX15 contributes to joint destruction through two interconnected mechanisms: it acts both as a downstream effector of inflammatory cytokines and as a key executor of ferroptosis.

The pro-inflammatory function of ALOX15 is closely linked to TNF-α signaling. Studies have demonstrated that ALOX15 mediates the upregulation of matrix metalloproteinases (MMPs) in RA synovial fibroblasts in response to TNF-α stimulation, thereby triggering chronic inflammation and promoting the degradation of bone and cartilage (159–161). This positions ALOX15 as a critical amplifier of cytokine-driven tissue damage. Building on this, further mechanistic studies revealed that the synovial microenvironment in RA—characterized by iron overload and oxidative stress—activates p53 signaling, which in turn upregulates ALOX15 expression. Elevated ALOX15 directly initiates uncontrolled lipid peroxidation chain reactions, inducing ferroptosis in synovial cells, chondrocytes, and other joint-resident cells. The resulting loss of cell membrane integrity not only exacerbates joint tissue destruction but also releases damage-associated molecular patterns that perpetuate local inflammation (162).

The therapeutic relevance of targeting this hub is underscored by recent findings with the P2Y12 inhibitor ticagrelor. Wu et al. (163) demonstrated that ticagrelor exerts anti-inflammatory and joint-protective effects precisely through modulation of the p53/SLC7A11/ALOX15 axis, significantly inhibiting both the expression and enzymatic activity of ALOX15. This intervention simultaneously dampens inflammatory mediator production and suppresses ferroptosis, highlighting the advantage of targeting a central node that governs multiple pathogenic pathways.

In summary, ALOX15 plays a central role in joint destruction in RA by integrating ferroptosis and inflammatory signaling and has emerged as a promising target for therapeutic intervention.

#### 3.4.2 Inflammatory bowel disease

ALOX15 plays a complex and critical role in the pathogenesis, progression, and therapeutic response of inflammatory bowel disease (IBD). Its pathological functions are primarily manifested in the following aspects: Firstly, ALOX15 is a key molecule driving intestinal inflammation and tissue damage in IBD. Studies have confirmed that in dextran sulfate sodium (DSS)-induced ulcerative colitis organoid and mouse models, ALOX15 was a crucial regulatory gene for ferroptosis. Its upregulation facilitated ferroptosis within intestinal epithelial cells, thereby contributing to the dismantling of the intestinal mucosal barrier, disrupting crypt architecture, and exacerbating inflammatory responses. Notably, knocking down ALOX15 significantly mitigated the progression of colitis, as demonstrated in study (164). Concurrently, ALOX15 serves as a crucial bridge linking chronic inflammation to carcinogenesis. In colitis-associated tumorigenesis (CAT) models, ALOX15 has been identified as one of the potential oncogenes. Its expression was upregulated during tumor development and correlated with elevated levels of pro-inflammatory cytokines such as TNF-α and IL-6. Inhibition of CD73 downregulated ALOX15 and attenuated tumorigenesis, whereas activation of adenosine receptors upregulated ALOX15 and exacerbated tumor development. These findings suggest that ALOX15 plays a pivotal role in the microenvironmental regulation of IBD-associated carcinogenesis, highlighting the intricate interplay between inflammatory signals and tumorigenic processes (165).

However, its function exhibits metabolic context-dependence. On one hand, in the context of classical n-6 PUFA metabolism (e.g., AA), ALOX15 may generate pro-inflammatory mediators that exacerbate inflammation. On the other hand, in a setting of elevated tissue levels of n-3 PUFAs, ALOX15 catalyzes the production of potent anti-inflammatory lipid mediators, thereby mediating the protective effects of n-3 PUFAs. Genetic deletion of Alox15 completely abolishes this protection, highlighting its indispensable role in mediating inflammation resolution (166).

Furthermore, Alox15 holds significant clinical relevance. Its dynamic expression can serve as an early predictive biomarker for the efficacy of anti-TNF therapy, such as infliximab. In the blood of IBD patients who have not achieved therapeutic remission, the continual high expression of ALOX15 and genes associated with Th2/eosinophil activity may indicate an immune state that is not conducive to a favorable treatment response. A lack of associated changes in gene expression and methylation signatures early in treatment may predict the patient’s primary non-response to TNF antagonists (167). Therefore, ALOX15 emerges as a pivotal element, integrating the roles of detrimental factor, environmental sensor switch, prognostic indicator, and potential drug target in IBD. This makes it crucial for comprehending disease mechanisms and formulating innovative therapeutic strategies.

### 3.5 Metabolic inflammatory diseases

#### 3.5.1 Type 2 diabetes

In the pathogenesis and progression of type 2 diabetes, the role of ALOX15 permeates multiple critical aspects including glucose and lipid metabolism disorders, chronic inflammation, and multi-organ damage. At the level of metabolic organs, the upregulation of ALOX15 expression is a significant mechanism underlying diabetic fatty liver disease and adipose tissue dysfunction. In the liver, ALOX15 activates the PPARγ/CD36 pathway by its metabolites, markedly enhancing fatty acid uptake and directly leading to hepatocyte lipid accumulation and lipotoxicity (168). Concurrently, in visceral adipose tissue, increased ALOX15 expression is closely associated with local inflammation. The pro-inflammatory lipid mediators recruit and activate macrophages, exacerbating adipose tissue inflammation and thereby driving systemic insulin resistance (169).This inflammatory spillover effect similarly impairs pancreatic islet function. Research indicated that ALOX15 promoted macrophage infiltration into pancreatic islets and exacerbated the “dedifferentiation” (loss of function) process in β-cells, directly weakening insulin secretory capacity (170). Clinical genetic data further support its importance in human disease, emphasizing the role of the lipoxygenase-inflammatory axis in disease susceptibility (171).Its pathological impact even extends to diabetic complications. In diabetic nephropathy, ALOX15 is synergistically upregulated with the ferroptosis-driver protein HMOX1, and their levels positively correlate with renal inflammation markers, suggesting their potential involvement in kidney injury by promoting programmed cell death (172).

Intervention studies targeting ALOX15 provide strong evidence supporting the aforementioned mechanisms. In animal models, both the use of a selective human ALOX12/15 inhibitor (VLX-1005) and the specific knockout of the ALOX15 gene in macrophages significantly improved hyperglycemia, reduced inflammation in adipose tissue and pancreatic islets, and decreased β-cell dedifferentiation (170). Furthermore, the plant-derived natural compound corilagin has been shown to simultaneously inhibit α-glucosidase and ALOX15 (173). Classical glucose-lowering agents such as liraglutide (GLP-1 receptor agonist) and metabolic surgeries (e.g., duodenal-jejunal bypass) have also been found to improve hepatic lipid metabolism, with their mechanisms of action partly involving the inhibition of the ALOX15/PPARγ/CD36 pathway (174). These findings collectively demonstrate that ALOX15 is a highly promising, pleiotropic therapeutic target, offering a clear direction for developing novel therapies that simultaneously ameliorate metabolic disorders and chronic inflammation.

#### 3.5.2 Non-alcoholic fatty liver disease

In the complex pathological process of non-alcoholic fatty liver disease (NAFLD), disruption of iron homeostasis and ferroptosis play a central driving role. Among these, ALOX15, as a key executor of ferroptosis, constitutes a core hub linking lipid metabolism dysregulation, oxidative stress, and programmed hepatocyte death (175). Studies have shown that under lipotoxic and iron-overloaded conditions in NAFLD, ALOX15 is significantly activated and cooperates with upstream molecules—acyl-CoA synthetase long-chain family member 4 (ACSL4) and lysophosphatidylcholine acyltransferase 3 (LPCAT3)—to form the “ACSL4/LPCAT3/ALOX15” axis. Which specifically catalyzes lipid peroxidation in cell membranes. This process not only disrupts membrane integrity, leading to ferroptosis in hepatocytes, but the released damage-associated molecular patterns (DAMPs) further exacerbate hepatic inflammatory infiltration and stellate cell activation, thereby driving the progression from simple steatosis to NASH, liver fibrosis, and even hepatocellular carcinoma (175, 176).

Multiple studies collectively supported this central role of Alox15: the ferroptosis inhibitor Liproxstatin-1 alleviated metabolic-associated fatty liver disease in mice by inhibiting ALOX15 (177). A network pharmacology analysis revealed that the anti-NAFLD effect of Delphinium brunonianum extract was closely related to the regulation of ALOX15 (178). Diosgenin reduced hepatocyte lipid accumulation by modulating the “ACSL4/LPCAT3/ALOX15” pathway (176). And the protective effect of zeaxanthin also involved downregulating ALOX15 expression to suppress lipid peroxidation (179). Thus, ALOX15 not only serves as a biomarker of ferroptosis in NASH but also acts as a key effector driving the vicious cycle of hepatocyte injury, inflammation, and fibrosis, making it a highly promising therapeutic target.

All the roles of ALOX15 in inflammation-related diseases were summarized in **Table 3**.

**Table 3:** The role of ALOX15 in inflammatory diseases.

Disease	Core role	Key mediators	Main effects	References
Nervous system				
Ischemic brain	Pro-inflammation Pro-injury	12(S)-HETE 15(S)-HETE	Promote oxidative stress and lipid peroxidation. Damage the blood-brain barrier and aggravate brain edema. Chemotactic neutrophil infiltration. Directly involved in the neuronal death pathway.	(90–93)
	Anti-inflammatory Restorative	LXA4, NPD1	Inhibit microglial activation and neutrophil infiltration. Alleviate inflammation and reduce infarct area. Maintain the integrity of the blood-brain barrier and reduce edema. Promote neuroprotection and the resolution of inflammation.	(22, 96–99)
AD	Pro-inflammation Pro-pathology	12(S)-HETE 15(S)-HETE	Promote the formation of Aβ plaques. Promote excessive phosphorylation of tau protein. Aggravate oxidative stress and neuroinflammation. leads to synaptic dysfunction and memory impairment.	(101, 102, 105, 106)
PD	Pro-inflammation Induce ferroptosis	15(S)-HpETE 12(S)-HpETE	Response to oxidative stress, trigger neuronal death. Mediating neurotoxicity conversion. Driving ferroptosis.	(53, 111–113, 116)
NP	Pro-inflammation Pro-injury Induce pain	15(S)-HpETE 12(S)-HpETE Hepoxilin	Increase lipid peroxidation. aggravate neuroinflammation. Driving ferroptosis. Induce and maintain hypersensitivity to pain.	(119–121, 128, 180)
Cardiovascular system				
Atherogenesis	Pro-inflammation pro-atherosclerosis	15(S)-HETE ox-LDL ox-HDL	Damage the endothelial barrier. Drive the formation of foam cells. Aggravate inflammation.	(122, 124, 126, 132, 137, 138)
	Anti-inflammatory Anti-atherosclerotic	13(S)-HODE LXA4, RvD1	Promote the outflow of cholesterol. Promote the resolution of inflammation.	(134, 141, 142)
MIRI	Pro-injury	15-HpETE 12-HETE 15-HETE	Intensify oxidative stress. Amplify the inflammatory response. Trigger ferroptosis.	(144–147)
Respiratory system				
Asthma	Pro-inflammation Pro-injury	15-HpETE 12-HETE 15-HETE	Drive ferroptosis of airway epithelial cells. Intensify the inflammatory response.	(150, 152)
	Anti-inflammatory Promote regression	RvD1, MaR1	Inhibit eosinophil infiltration and relieve airway inflammation. Promote the resolution of inflammation.	(153, 154)
COPD	Pro-inflammation Pro-injury	15-HpETE 12-HETE 15-HETE	Ferroptosis occurs in the epithelial cells of the bronchi and alveoli. Maintain vicious cycle of chronic inflammation.	(15, 156)
Autoimmune diseases				
RA	Pro-inflammation Pro-injury	15-HpETE 12-HETE 15-HETE	Drive ferroptosis of joint tissues. Promote chronic inflammation and the destruction of bones and cartilage.	(159, 162)
IBD	Pro-inflammation Pro-injury	15-HpETE 15-HETE 12-HETE	Drive ferroptosis of intestinal epithelial cells, damages the mucosal barrier and aggravates inflammation. Promote tumorigenesis.	(164, 165)
Metabolic diseases				
Type 2 diabetes	Pro-inflammatory Pro-metabolic disorder	12-HETE 15-HETE	Drive hepatic steatosis. Aggravate inflammation of adipose tissue. Damage pancreatic islet function. Participate in diabetic nephropathy injury.	(168–170, 172)
NAFLD	Pro-inflammation Pro-injury	15-HpETE 15-HETE 12-HETE	Drive ferroptosis of hepatocytes. Promote vicious cycle of inflammation and fibrosis.	(175, 176)

LXA4, lipoxinA4; NPD1, neuroprotection D1; AD, Alzheimer’s disease; PD, Parkinson’s disease; NP, neuropathic pain; RvD1, resolvin D1; ox-LDL, oxidize low-density lipoprotein; ox-HDL, oxidize high-density lipoprotein; MaR1, maresin 1; MIRI, myocardial ischemia-reperfusion injury; COPD, chronic obstructive pulmonary disease; RA, rheumatoid arthritis; IBD, inflammatory bowel disease; NAFLD, non-alcoholic fatty liver disease.

## 4 The therapeutic strategies targeting ALOX15

### 4.1 Small molecule inhibitor

A growing number of small molecule inhibitors targeting ALOX15 have been developed and characterized in recent years, exhibiting varying degrees of potency, selectivity, and therapeutic potential across different disease models (181). These inhibitors can be broadly categorized based on their target specificity and mechanism of action.

Among the most well-characterized inhibitors, PD146176 is a selective competitive inhibitor of ALOX15 that has demonstrated efficacy in models of vascular calcification, renal fibrosis, and inflammation (181, 182). ML351 represents another highly selective inhibitor, displaying over 100-fold selectivity for ALOX15 compared to ALOX5 and ALOX12, and has shown neuroprotective effects in ischemic stroke models by attenuating glutamate oxidative toxicity (181). These inhibitors exhibit high selectivity, with minimal effects on other members of the lipoxygenase family, thereby offering relatively controllable side effects (183, 184).

The flavonoid baicalein and its prodrug baicalin are frequently used ALOX15/12 dual inhibitors that reduce lipid peroxidation and iron accumulation, exhibiting protective effects in neurodegenerative disorders and ischemia-reperfusion injury (181, 182). In contrast, NDGA (nordihydroguaiaretic acid) is a broad-spectrum lipoxygenase inhibitor with redox activity, but its lack of selectivity limits its utility as a specific tool compound (181). While these natural inhibitors generally show pleiotropic effects and favorable safety profiles, their potency and specificity are often lower compared to synthetic compounds (87, 185).

Recent drug discovery efforts have also yielded highly potent inhibitors with improved selectivity. Patent compound WO0196298, a 1,2,4-trisubstituted benzene derivative, exhibits an IC50 as low as 5nM against human ALOX15 with excellent selectivity (186). Additionally, a new class of inhibitors targeting the ALOX15/PEBP1 complex—including isochlorogenic acid C and FerroLOXINs—has emerged as a strategy to specifically interfere with ferroptosis execution without broadly inhibiting all ALOX15 enzymatic activities (182). It is important to note that inhibitor specificity can vary significantly between human and mouse orthologs, and compounds validated in human systems may exhibit different potency profiles in mouse models (187).

Multi-target inhibitors enhance anti-inflammatory efficacy by synergistically inhibiting ALOX15 along with other key enzymes in inflammatory pathways. In addition to ALOX15, these multi-target inhibitors (e.g., CDC) have been shown to inactivate ALOX12 and hepoxilin synthase (HXS) activity, including that mediated by ALOXE3, thereby suppressing the production of pro-inflammatory mediators such as hepoxilins and 12-HETE, both of which contribute to neuroinflammation and pain hypersensitivity. This strategy is particularly suitable for complex inflammatory conditions where “escape pathways” of lipid mediators exist (188).

**Table 4** summarizes the key characteristics of representative ALOX15 inhibitors discussed above, providing a reference for selecting appropriate tool compounds based on specific research needs.

**Table 4:** Representative small molecule inhibitors targeting ALOX15 and their characteristics.

Inhibitor	Target specificity	Mechanism of action	Disease models	References
PD146176	ALOX15	Competitive inhibition	Vascular calcification, renal fibrosis	(181, 182)
ML351	ALOX15	Imidazole-based inhibitor	Ischemic stroke	(181)
Baicalein	ALOX12/15	Flavonoid, reduces lipid peroxidation	Neurodegeneration, stroke	(181, 182)
NDGA	Pan-LOX inhibitor	Redox inhibitor, antioxidant	Diabetic nephropathy	(181)
Compound (WO0196298)	ALOX15	1,2,4-trisubstituted benzene	Inflammation, atherosclerosis	(186)
Isochlorogenic acid C	ALOX15/PEBP1 complex	Disrupts complex formation	Ferroptosis	(182)
FerroLOXINs	ALOX15/PEBP1 complex	Targets the enzyme complex	Ferroptosis	(182)

NDGA, Nordihydroguaiaretic acid; PEBP1, Phosphatidylethanolamine binding protein 1.

### 4.2 Gene regulation strategy

Gene regulation strategies focus on intervening in ALOX15 expression at the source, including: (1) Downregulation of mRNA using siRNA or antisense oligonucleotides. For example, local delivery of ALOX15 siRNA to the airways of asthmatic mice significantly reduced eosinophil counts and Th2 cytokine levels in bronchoalveolar lavage fluid (152). In atherosclerosis models, systemic administration was able to reduce plaque area and lipid core size. Advances in nano-delivery systems are progressively addressing the targeting and stability challenges of such strategies (189). (2) Knockout of the ALOX15 gene in specific cell types using CRISPR/Cas9 technology can fundamentally block the production of its pro-inflammatory metabolites (190). Although *in vivo* gene editing still faces challenges in delivery efficiency, off-target effects, and ethical considerations, it represents a conceptual breakthrough for genetic or chronic inflammatory diseases. (3) Modulation of expression via epigenetic drugs, such as protein deacetylase inhibitors, can alter the promoter activity of ALOX15, downregulating its expression. In inflammatory models, these drugs broadly regulate multiple inflammation-related genes, and combine with ALOX15 inhibitors may produce synergistic effects (191).

### 4.3 Metabolic reprogramming

Metabolic reprogramming regulates the ALOX15 pathway through indirect approaches. For instance, increased intake of omega-3 PUFAs (e.g., EPA and DHA) can competitively reduce utilization of omega-6 substrates (e.g., AA). This shift in production towards anti-inflammatory mediators like RVs and PDs instead of pro-inflammatory HETEs. Epidemiological and clinical trial data suggest that omega-3 PUFAs supplementation provides auxiliary benefits in managing cardiovascular diseases and asthma, partly mediated through this mechanism (192–194). Alternatively, enhancing the lipid peroxide clearance system can block downstream inflammatory responses. For example, activating Nrf2 to upregulate GPX4 activity promptly reduces the hydroperoxides generated by ALOX15, preventing their further conversion into active mediators or ferroptosis (195). Furthermore, developing compounds that specifically clear 15-HpETE-PE represents an emerging direction for intervening in ferroptosis-associated inflammation. However, these findings should be interpreted cautiously without independent validation from other loss-of-function and/or gain-of-function experimental strategies.

## 5 Challenges and prospects

Although targeting ALOX15 holds promise for the treatment of inflammatory diseases, translating this potential into clinical applications faces substantial challenges that stem from the enzyme’s complex biology, the intricate lipid mediator network it modulates, and significant interspecies differences. Addressing these obstacles requires a multidimensional research strategy that integrates technological innovation, systems-level mechanistic understanding, and personalized therapeutic approaches.

### 5.1 Tissue specificity and disease-stage dependence

A fundamental challenge lies in the context-dependent duality of ALOX15. While it promotes inflammation and ferroptosis in certain settings—such as in atherosclerotic plaques (136) or arthritic synovium (158)—it also generates pro-resolving mediators that actively terminate inflammation and promote tissue repair (153, 154). This functional plasticity means that systemic ALOX15 inhibition, while potentially beneficial during active inflammation, might inadvertently disrupt resolution phases or compromise homeostatic functions in unaffected tissues. For instance, global Alox15 knockout in mouse models has yielded opposing outcomes depending on the disease stage examined (141, 142), underscoring the need for temporally and spatially refined interventions.

### 5.2 Complexity of the lipid mediator network

ALOX15 is embedded within a vast and interconnected network of lipid metabolic pathways. Inhibiting ALOX15 alone may perturb the balance of other bioactive lipids, potentially triggering compensatory “shunting” to alternative pathways—a phenomenon well-documented in the eicosanoid field (196). For example, pharmacological inhibition of ALOX5 can redirect arachidonic acid metabolism toward ALOX15 pathways, and vice versa (197). Such metabolic escape could not only diminish therapeutic efficacy but also generate unanticipated off-target effects. A comprehensive understanding of these network-level adaptations is essential for predicting the consequences of ALOX15 modulation.

### 5.3 Drug delivery and selectivity bottlenecks

Achieving cell-type-specific and site-selective targeting remains a major technical hurdle. ALOX15 is expressed in multiple cell types—including macrophages, epithelial cells, and endothelial cells—each contributing differently to disease pathogenesis (30). Systemic delivery of ALOX15 inhibitors may thus produce mixed or opposing effects by simultaneously targeting cells with distinct functional roles. Recent advances in nanomedicine offer potential solutions: nanoparticle-based delivery systems functionalized with cell-specific ligands have shown promise for targeted delivery to inflamed tissues (198), and prodrug strategies that exploit disease-associated enzymatic activity for local activation are under active investigation (199).

### 5.4 Interspecies differences

The translation of preclinical findings to humans is complicated by significant interspecies variation in ALOX15 biology. Human and rodent ALOX15 exhibit distinct substrate preferences and catalytic properties. For instance, human ALOX15 preferentially oxygenates arachidonic acid to 15-HpETE, whereas 12-HpETE is the dominant oxygenation product of the mouse Alox15 ortholog (24, 25). This functional divergence stems from structural variations in the substrate-binding pocket, with a single amino acid residue (Phe353 in humans vs. Leu353 in mice) playing a critical role in determining reaction specificity (200). Beyond catalytic properties, expression patterns and regulatory mechanisms may also differ between species, cautioning against direct extrapolation of knockout or inhibitor studies from rodents to humans (201, 202).

Recent advances in generating “humanized” Alox15 knock-in mice, which express a mutant enzyme (Leu353Phe) with human-like 15-lipoxygenating activity, have begun to address this gap. Studies using such models have revealed that humanization of Alox15 confers protection in dextran sodium sulfate-induced colitis, suggesting that the human enzyme may possess enhanced pro-resolving capacity (203). These findings underscore both the importance and the feasibility of accounting for species differences in preclinical drug development.

### 5.5 Future directions

Addressing these challenges will require a coordinated, multi-pronged research agenda. First, technological innovation should focus on developing precision delivery systems—such as lipid nanoparticles or exosome-based carriers—that enable cell-type-specific ALOX15 modulation. Second, systems-level mechanistic studies employing multi-omics approaches (lipidomics, transcriptomics, proteomics) are needed to map the dynamic lipid mediator networks in which ALOX15 operates across different disease contexts and stages. Such integrated analyses could identify predictive biomarkers of therapeutic response and reveal optimal windows for intervention. Third, personalized medicine strategies should be explored, leveraging genetic polymorphisms in the ALOX15 gene that may influence enzyme activity or substrate preference to stratify patients and tailor treatment regimens. Finally, temporal intervention strategies—adjusting therapy based on disease phase (e.g., acute inflammation vs. resolution)—may harness the dual functions of ALOX15, selectively inhibiting its pro-inflammatory actions while preserving or even enhancing its pro-resolving activities. Ultimately, a deeper understanding of the contextual regulation of ALOX15 will be essential to unlock its full therapeutic potential.

## 6 Conclusion

ALOX15 serves as a pivotal nexus connecting lipid metabolism and inflammatory responses, playing a significant role in various inflammatory diseases. Therapeutic strategies targeting ALOX15 demonstrate broad potential, although they also face complex challenges. Future research necessitates a deeper understanding of its precise function within specific disease contexts, the development of spatiotemporally specific regulatory tools, and the exploration of synergistic effects with other treatment approaches. Multi-target regulatory strategies informed by a systems biology perspective, particularly those leveraging the synergistic actions of multi-component natural products, may offer novel pathways for the treatment of complex inflammatory diseases.
